# Biomechanical Evaluation of Recurrent Dissociation of Modular Humeral Prostheses

**DOI:** 10.3390/bioengineering9020076

**Published:** 2022-02-16

**Authors:** Daniel B. Luckenbill, Mike F. Iossi, Alyssa M. George Whitney, Danielle Miller, Lynn A. Crosby, Tarun Goswami

**Affiliations:** 1 Boonshoft School of Medicine, Wright State University, Dayton, OH 45435, USA; luckenbill.3@wright.edu; 2 Department of Biomedical Engineering, Orthopaedic Surgery, Sports Medicine and Rehabilitation, Wright State University, Dayton, OH 45435, USA; mfiossi@yahoo.com (M.F.I.); alyssa.whitney@zimmerbiomet.com (A.M.G.W.); miller.612@wright.edu (D.M.); 3 Department of Orthopedic Surgery, Medical College of Georgia, Augusta, GA 30912, USA; lycrosby@augusta.edu

**Keywords:** modular prostheses, dissociation of humeral head, shoulder arthroplasty, torque to dissociation, recurrent dissociation

## Abstract

The purpose of the study was to evaluate the force and torque required to dissociate a humeral head from the unimplanted modular total shoulder replacement system from different manufacturers and to determine if load and torque to dissociation are reduced in the presence of bodily fluids. Impingement, taper contamination, lack of compressive forces, and interference of taper fixation by the proximal humerus have all been reported as possible causes for dissociation. Experimental values determined in this research were compared with literature estimates of dissociation force of the humeral head under various conditions to gain more understanding of the causes of recurrent dissociations of the humeral head. This study examined biomechanical properties under dry and wet conditions under clinically practiced methods. Mean load to dissociation (1513 N ± 508 N) was found to be greater than that exerted by the activities of daily living (578 N) for all implants studied. The mean torque to dissociation was (49.77 N·m ± 19.07 N·m). Analysis of *R*^2^ correlation coefficients and *p*-values (α = 0.05) did not show any significant correlation between dry/bovine, dry/wet, or wet/bovine for load, displacement, or torsional stiffness in the majority of tests performed. Wetting the taper with water or bovine serum did not reduce the dissociation force to a statistically significant degree. Torque and lack of compressive forces at the rotator cuff may be the cause of dissociation at values less than those of activities of daily living. Torque data are provided by this study, but further research is needed to fully appreciate the role of torque in recurrent dissociations.

## 1. Introduction

Motivation for this study was a case report of a 67 years old male with recurrent dissociation of a modular humeral prosthesis following hemiarthroplasty for a four-part humerus fracture. Most humeral head dissociations [1,2,3,4,5,6,7,8,9,10,11,12,13,14,15,16,17,18,19,20,21,22] occur during activities of daily living (ADLs), which is of interest since the average force to dissociate a humeral head from components is much higher than would be expected during ADLs. Physiologic ADL values are difficult to assess and can be determined using various models [3]. Charlton and Johnson tested 10 different ADLs and determined the peak force from ADLs to be 577.6 N [3]. The present study uses this value to represent a best available value for forces at the glenohumeral joint since the data reported therein were from the human cadavers and not computationally determined. Accurate in vivo load parameters are not available; therefore, the present study assumes values obtained from cadavers.

A modular component is defined as one that is assembled at the time of surgery [14]. Modular humeral prostheses offer several advantages over the original monoblock design that was introduced in 1955 by Dr. Charles Neer II [15]. Modular implants use a Morse taper design, although with a larger angle (4–6°), and with a shorter socket and shank length [1]. The Morse taper is designed to achieve fixation through friction. Friction between the two components holds the modular head component in place until a force, either “pull” or rotational, overcomes the coefficient of friction and begins to loosen the fixation. Friction is commonly used for fixation in orthopedics trauma instrumentation, and one such instance was described by Goswami et al. [8] who analyzed locking compression plates used in open reduction and internal fixation of fractures. In their study, they found that fixation remained stable until the axial force exceeded the frictional force at which time the component began to loosen. Similarly, when various factors outlined in this study result in a decrease in the coefficient of friction at the Morse taper, repetitive forces of activities of daily living may result in a loss of fixation. Advantages of the modular design and proper postoperative radiographic evaluation have been well described in the literature [7,11,13,17,21].

Shoulder arthroplasty has been described as a difficult joint procedure [20]. Several complications can arise such as loosening of components, dislocation, rotator cuff tears, infection, and dissociation of modular components. The overall rate of complications following a total shoulder arthroplasty is reported in the literature to be 10–15% [22]. Dissociation of a modular humeral head has been reported to occur at an incidence of one per 1000 [1]. In order to biomechanically determine glenohumeral force to dissociate a well-positioned, compressed humeral head, tension loads are applied. The load at which the humeral head dissociates allows measuring the compressive forces of the Morse taper assembly. In addition to the axial measure of forces, physiological ranges of motion and appropriate torque developed may play a role in dissociation of the humeral heads. Therefore, the objective of this paper was to undertake an experimental study by simulating the operating room procedures to assemble the shoulder joint and determine load to dissociate in tension independently and torque to dissociate in the presence of dry and bodily fluids. 

Biologic debris and/or fluid in the socket of the Morse taper have been implicated as a cause of in vivo disassembly [1,11,17]. Micromotion at the taper junction during regular use has been mentioned in the literature as a cause of wear debris to form in the taper and is a potential cause of loosening; however, the majority of recurrent dissociations reported in the literature occur early (approximately 6 weeks) following surgery [1,5], which suggests that micromotion at the taper junction producing wear debris is not likely a cause of recurrent dissociation. Instability due to insufficient muscle tension has been demonstrated to lead to recurrent dissociation of modular prostheses [5].

Results of total shoulder arthroplasty would suggest that outcome is not sacrificed for convenience when modularity is employed. Evaluation of 34 monoblock and 34 modular total shoulder arthroplasties done for osteoarthritis demonstrated no significant differences in clinical outcome or radiological changes [5]. Nevertheless, use of modular implants is associated with a few unique potential complications, one of which is dissociation of the humeral stem and head.

We are aware of 16 reported cases of humeral head–stem dissociation [1,5,19,23]. In these cases, possible causes for dissociation were reported to be impingement [5], taper contamination [1], lack of compressive forces [19], and interference of taper fixation by the proximal humerus [23]. While the dissociation mechanisms are defined in terms of impingement, taper contamination, and interference, the focus of this study was to determine whether or not there is a sufficient amount of compression forces present at the junction and torque to dissociate retrieved implants, as well as to compare the biomechanical parameters of retrieved implants with unused implants.

To our knowledge, no other study to date has compared four major manufacturers of modular shoulder prostheses in a single study to accepted ADL values under a range of circumstances. In addition, this study assessed torque to dissociation, which has not been previously studied and contributes to the physiological understanding of glenohumeral joint (GHJ). The results of this study may benefit orthopedic surgeons in preventing dissociation events, as well as recognizing some of the features that may make a patient less suitable for modular type prosthesis. The objective of the present study was to evaluate the load and torque to dissociation of humerus heads obtained from revision surgeries as retrieved implants, and to determine if load and torque to dissociation are reduced by the presence of bodily fluids.

## 2. Materials and Methods

Biomechanical testing to determine head dissociation was carried out by pulling the shoulder assembly from the mechanical testing machine EnduraTech (Minnetonka, MN, USA). The assembly was performed by a graduate biomedical engineering student who followed the clinical specifications regarding mallet angle (45°) and number of strikes (two strikes). Nine retrieved shoulder implants and one implant (Exactech, Gainesville, FL USA) obtained from the manufacturer were assembled 10 times each, and the results were documented. The group consisted of five Zimmer, two Biomet, one DePuy, and two Exactech implants. Ten trials of manual impaction were performed followed by controlled distraction of the implant. This system was a 12.6 kN axial/torsion test frame. The force at the time of dissociation was recorded which measured compression/friction forces present at the Morse taper. The sequence was repeated 10 times for each of the 10 implants, totaling to 100 tests.

**Figure A1:**
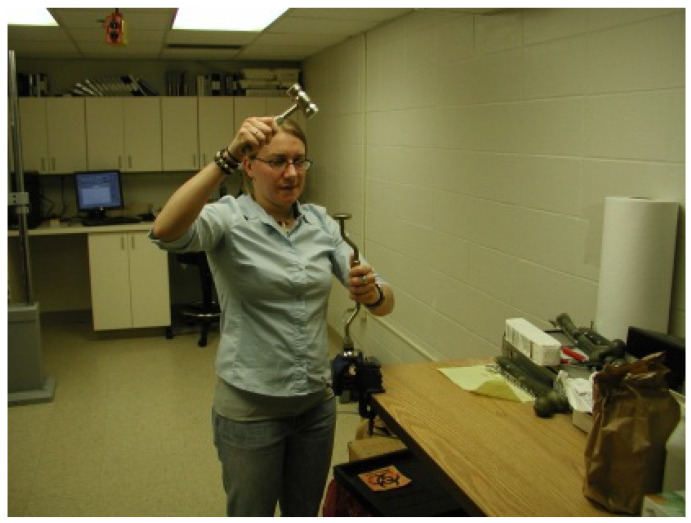
Experimental setup to prepare the glenohumeral implant assembly.

Assembly of glenohumeral joint was made by fixing the stem to a vice grip with the taper shank oriented perpendicular to the floor. The head component was placed on the stem, and two impacting blows were delivered using a head impactor and mallet. With the vice grip and stem at approximately waist level, the mallet was raised with one hand to approximately ear level. The blows were delivered manually with an attempt to reproduce the motion and force of impaction for each blow. Two blows were used following the literature [10,22] and clinical advice obtained in the laboratory. This study used manual impaction as is the case in an operating room, while other studies used machine impaction in an attempt to better simulate a mechanically prepared joint. However, such an assembly also has effects due to the alignment of stem and head, as well as the rate at which forces are applied and the magnitude of forces applied. The laboratory setup to prepare the glenohumeral joint assembly is shown in Appendix A, Figure A1.

The component stem was then fixed to the vice grip of the machine with the taper shank or female end compressing the head between two flat surfaces in the grips with the plane of compression oriented parallel to the floor. It was assumed that this compression would not alter the structure of the head taper and, therefore, not affect the mechanical properties of fixation.

With the implant secured to the machine, an axial distraction force was applied. The applied load was measured using an EnduraTEC 2.2 kN axial/torsion biaxial load cell (Model No. 1215CEW-250). The Win Test Digital Control System (EnduraTEC Minnetonka, MN, USA) was used to determine the input parameters and monitor the output parameters. Axial testing was performed in displacement control mode, while torsional testing was done in rotation control mode. The maximum displacement occurred at the point of maximum rotation. Input waveforms for both modes were ramp waveforms. In tension mode, the load changed from an initial negative value to a positive value. The data acquisition system scanned the test parameters at every 0.1 s; thus, the ramp rates were accurately controlled.

Ten force-to-dissociation data points for each of the 10 implants were recorded. The mean standard deviation and 95% confidence interval were calculated for each implant. To detect statistically significant differences, all possible pairwise differences were examined using the least square difference (LSD) post hoc comparison. These data were then compared to previous studies of biomechanical pull-off strength and literature values for forces across the glenohumeral joint during ADLs. The testing program used in this research was consistent with literature for the axial loads to dissociate the implants. All the experimental data comprising the mean and standard deviation for 10 tests are presented in Appendix A, Table A1.

Since dissociation of the humeral head from the stem is a rare event, the total number of such events is very low. A viable statistical study design is, therefore, not possible since the number of samples needed for testing would be very high to provide a high enough power (95%). Therefore, such a study would require a national-level effort to obtain each retrieved device for testing. This is also very difficult since retrieved devices are taken by the patients in most cases and they may opt to not participate in the research. Therefore, our statistical analyses used laboratory-generated data for each sample tested 10 times. Therefore, the total number of tests was 100 in this study. Statistical analysis of our torque, rotation, load, and displacement data was performed for different implants with significance set at *p* < 0.05. Continued removal of the implant with the highest *p*-value was completed to achieve significant correlation. 

## 3. Results

No individual implant had a mean load to dissociation lower than 1000 N. The range for all implant trials (*n* = 100) was 723–2730 N. The mean load to dissociation across all implants (*n* = 10) was 1513 N. The mean load to dissociation was highest for implant 9 and lowest for implant 4. The maximum reported load during ADLs is 578 N, which is considerably less than the lowest range in the 95% CI for all implants that we studied (Table 1).

**Figure 1:**
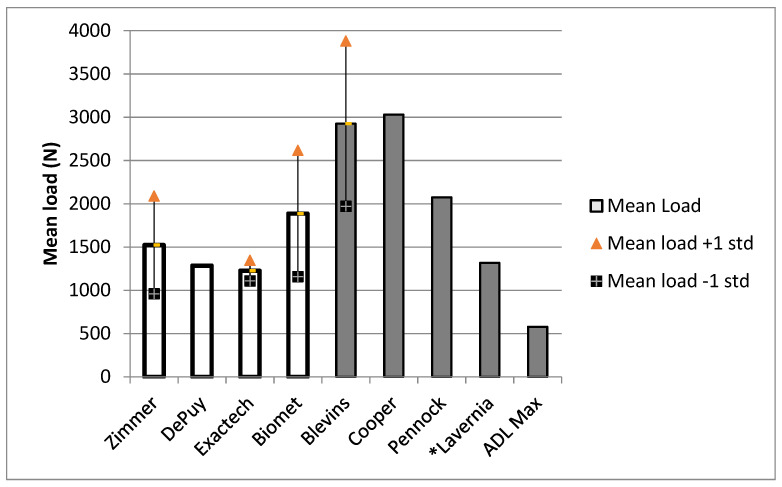
Mean load to dissociation compared to ADL max. *: The Lavernia data in Figure 1 represent an average of the mean dissociation forces across five trials of four different clean tapers.

Figure 1 compares the mean load to dissociation in this study (empty bars) to the literature values (filled bars) and the maximum reported ADL force. The mean loads to dissociation for the implants in our study were lower than those of the Blevins [1], Cooper [5], and Pennock [17] studies, but similar to that found for clean tapers in the Lavernia [11] study. This difference may be due to the impaction method used. Pennock et al. and Lavernia et al. [11,17] used drop towers in their studies, while Blevins et al. [1] used a MTS machine, as well as mallet impactions. This study used only mallet impactions in an effort to simulate operating room conditions. These forces were still higher than the max ADL load. One standard deviation below the mean was still higher than the ADL for all implants. Standard deviations were not available for the Cooper, Pennock, Lavernia, or ADL data; however, our data are presented in detail in Appendix A, Table A1.

Torque and rotation were found not to be significantly correlated. No significant correlation was found between all 10 implants and load. A significant correlation was found between a comparison of implants 2, 5, 6, 8, and 9 and load (*R*^2^ = 0.94, *p*-value = 0.0142). For displacement, observations in implants 1, 7, and 10 were found to be significantly correlated. ANOVA for torque to dissociation demonstrated no significant difference among the 10 implants (*p* > 0.05).

The Lavernia [11] data in Figure 1 represent an average of the mean dissociation forces across five trials of four different clean tapers. The mean load to dissociation was highest among the Biomet implants and lowest among the Exactech implants. The Biomet implants also had the highest variability in their load to dissociation (Table 2). The mean rotation and torsional stiffness were inconsistent across different units of the same brand for the Exactech and Biomet models, but fairly consistent across the Zimmer models. The Exactech implants exhibited both the highest and the lowest mean for rotation and torsional stiffness (Table 3).

The Zimmer implants consistently required the greatest amount of torque to dissociate the head components of those implants. The Biomet implants performed similarly to the Zimmer implants, with the Exactech implants requiring the least amount of torque to dissociate their head components (Table 4).

**Figure 2:**
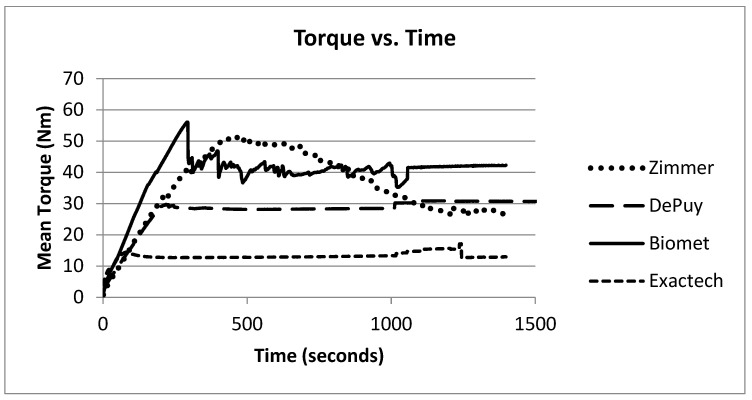
Mean torque vs. time for each implant.

Implants 1 and 2 began rotating before −30°; however, at the end of the test, implant 1 was easily removed with a screw driver and hammer, while implant 2 remained tight. Implants 3 and 4 began rotating before −30° and were easily pried off with screw drivers. Implant 5 was the only one of the 10 implants that did not rotate. Gouge marks on the side of the head component indicated slippage. Implant 6 rotated before +10° and was not easily removed at the end of the trial. During trials on implant 7, the vice would not hold the stem well, leading to difficulty hammering the head on. Rotation began very early in the test with the rotation occurring at the portion fixed to the stem. During the last trial on implant 7, the head was able to be removed by hand. The head component of implant 8 could not be adequately secured and slipped from the vice grips on every attempt. Implant 9 rotated before −37° but was still tight at the end of the test. Implant 10 rotated before 0°, and the portion that rotated was the part that was fixed to the stem. The torque developed vs. time during these trials increased initially and then decreased (Figure 2).

**Figure 3:**
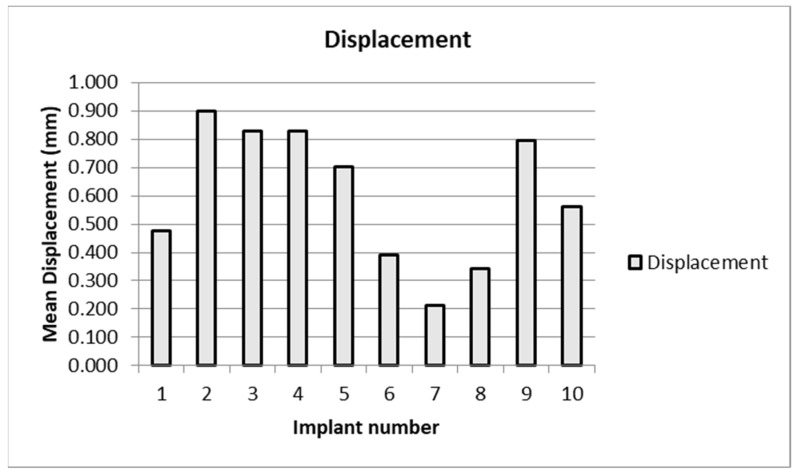
Mean displacement of each implant.

**Figure 4:**
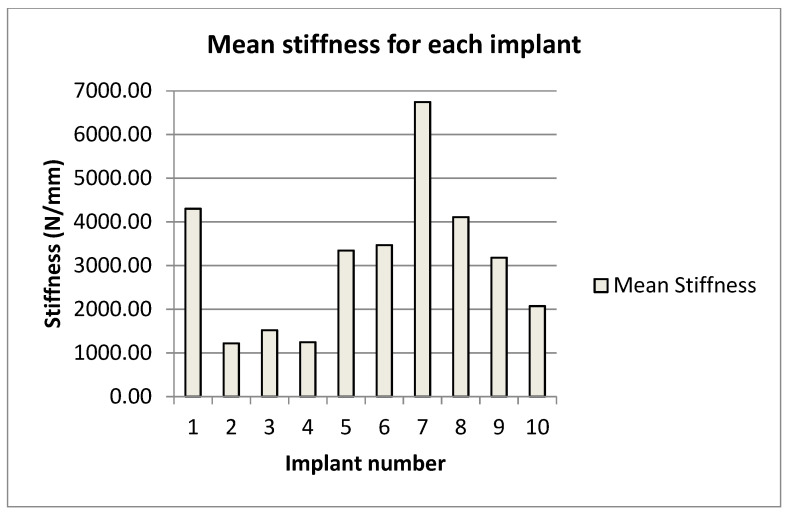
Mean axial stiffness compared to mean load for each implant.

The mean displacement was on average greatest for the Zimmer implants. The two Exactech implants were inconsistent, as were the two Biomet implants (Table 5). Mean displacement was inversely correlated with the torsional stiffness (Figure 3 and Figure 4).

## 4. Discussion

### 4.1. Taper Contamination

Analysis of *R*^2^ correlation coefficients and *p*-values (α = 0.05) did not show any significant difference between tests under dry and wet conditions using bovine fluid simulating bodily fluids, dry and wet conditions using water, or wet conditions using water and bovine combinations for load, displacement, or torsional stiffness in the majority of tests performed (Table 6). Our findings agree with those of Loch et al. [12] who found that wetting the taper did not weaken the junction. While multiple studies show that contamination of the taper reduces the pull-off strength, we do not believe that this is a likely cause of recurrent dissociation of the modular head component.

Micromotion and wear debris have been examined in the literature as a product of fretting and corrosion. These factors are not likely responsible for the cases of recurrent dissociation that we studied as they would take longer than the 6 weeks on the average time to dissociate. However, there may be a possibility of excessive compression forces between the polymer liner and humeral head causing brinelling, which pivots the head with the liner, causing dissociation by physiological torque. It may be noted that, in the orthopedic literature, there is no mention of brinelling occurring in the total joint replacement, which is a mechanical failure mode. The data reported in Table 4 are original and have not previously been reported in the literature.

In the case in question, great care was taken to clean and dry the taper–lock interface, as well as to ensure that no proximal humerus bone encroached upon the junction of the modular components, thereby impairing fixation. We firmly believe that neither of these issues was a source of the dissociation. With respect to the lack of compressive force as a possible etiology, the patient did not have rotator cuff pathology as in the case reported by Sisto et al. [19]. In addition, although the initial dissociation presented with greater and lesser tuberosity displacement, the second surgery reapproximated the tuberosities and should have restored any compressive force that may have been lacking for some period following the index procedure. The experimental program conducted in this study clearly shows that, in each of the 100 tests performed with 10 implants, none of the tests showed a lack of compression. Therefore, lack of compression would not be a potential dissociation failure mode.

### 4.2. Torque, Rotation, Load, and Displacement

Torque is of interest to consider because it was implicated in dissociation at the glenoid component in a study by Feldman and Bunker [6]. This study examined a Copeland total shoulder prosthesis which uses a polyethylene liner and a metal backing plate for the glenoid component. The polyethylene liner attaches to the metal backing plate with a taper design. Feldman and Bunker [6] found that increasing torsional stiffness at the shoulder, which can occur with an increased humeral head size, increases the transmission of forces to the glenoid, and sufficient torque applied here can cause the glenoid component to dissociate [6]. Wallace et al. [21] supported this idea in their study of glenoid dissociation noting that patients with any condition, such as Parkinson’s disease, that results in an increase in muscle tension should not be considered for a modular glenoid because they are disposed to glenoid dissociation due to the enhanced muscular imbalance [22]. Too large a humeral head generates excess torque and loading on the glenoid [2,9,10]. Logically, these forces would also be applied to the humeral head. To our knowledge, there is no other report of torque to dissociation in the literature. In a study comparing development of torque at the glenohumeral joint (GHJ) in competitive swimmers versus controls, McMaster et al. [13] described torque values during adduction, abduction, internal rotation, and external rotation. The average physiologic torque generated by the abovementioned movements as described in the McMaster study can be estimated at 44.61 ± 14.12 N·m. A comparison of torque to dissociation in the present study to physiologic estimates of torque generated at the GHJ using a two-sample *t*-test indicated that these values were not significantly different (*p* = 0.18) when all implants were considered together. Zimmer implants demonstrated a mean torque to dissociation of 62.03 ± 8.63 N·m that was significantly higher than the physiologic estimates of torque (*p* = 0.0). Depuy and Exactech implants demonstrated mean torque values lower than the physiologic estimate (*p* = 0.01 and *p* = 0.0, respectively). The mean torque to dissociation of the Biomet implants was not significantly different from the physiologic estimate of torque (*p* = 0.18). Although more research is indicated to clearly define the relationship between torque and recurrent dissociations of the modular humeral head components, the authors hypothesize that implants with values equal to or lower than the physiologic estimate of torque (44.61 ± 14.12 N·m) may be more likely to dissociate than implants with greater torque to dissociation values. There are no experimental data for torque on the humeral head during activities of daily living reported in the literature. When comparing the load to dissociation of the various implants to the maximum reported force of ADLs, it is evident that the mean load to dissociation (when properly assembled with clean tapers and sufficient impaction force) of all implants studied was greater than the maximum force of ADLs.

The ANOVA test of the four different brands of implants revealed that the Biomet implants had a significantly greater load to dissociation than the other brands (*p* = 0.00), and the Zimmer models had a significantly greater load to dissociation than the Dupuy and Exactech models (*p* = 0.01); however, there was no significant difference between the Dupuy and Exactech models in terms of mean load to dissociation (*p* = 0.59). Although the Biomet implant was the model that demonstrated recurrent dissociations in the case that motivated this study, the Biomet implants exhibited the highest mean load to dissociation of the implants that were studied. 

Design specifications of the Morse taper for each implant were considered proprietary information by the manufacturer and not available to the researchers; thus, we are unable to comment on such features that may have accounted for similarities or differences in the performance of each implant. We are not, therefore, able to comment on each taper angle and resulting biomechanical behavior.

### 4.3. Forces at the Glenohumeral Joint

It is difficult to assess forces attributed to a specific movement because compound movements, including sliding, rotation, and spinning [4], occur in the joint during many movements of the arm such as rotation of the humerus during abduction of the arm. Praagman et al. reported a maximum force of 437 N using the computerized Delft Shoulder Model [18]. Charlton and Johnson reported a maximum ADL force of 577.7 N while lifting a block designed to represent an everyday object to shoulder height [3].

Achieving a balance in stability, range of motion, and muscle tension may also reduce the torque applied to the humeral head and lessen its contribution to a possible dissociation of the head component, among other complications. Modular arthroplasty offers an advantage in this regard due to the wider availability of head sizes, allowing the surgeon to create the correct geometry for the individual patient. Care should be taken to ensure that the taper is clean and dry prior to assembly, and that impaction occurs in the axis of the taper and not off-axis blows. Off-axis blows reduce the impaction force [17]. Likewise, the humerus must be well secured prior to impaction to ensure the full force of the blow is delivered to the head [1].

## 5. Conclusions

The present study offers important information about the axial force to dissociation because it simulated operating room conditions during impactions and compression in the Morse taper. Strengths of this study include comparisons across multiple manufacturers, assessment of additional variables such as torque, and use of the more realistic approach mentioned above. No individual implant had a mean load to dissociation less than 1000 N, thereby ruling out the possibility of lack of compression in the joint. Data support the notion that the likelihood of dissociation is independent of implant selection. The mean distractive forces required to dissociate the well-seated prosthesis in this study are well above the predicted glenohumeral joint forces during ADLs (578 N). The considerable variability in the force to dissociate Biomet prostheses may in part support why instances of dissociation follow these implants; however, no biomechanical reasons can be proposed at this point. Considerable attention has been placed on the role of taper contamination as a cause of dissociation. Although there are conflicting data on the effect of a wet taper on distraction force, ensuring that the taper is dry prior to assembly will prevent it from contributing to dissociations in future operations. The torque and lack of compressive forces at the rotator cuff may also result in dissociation at values less than ADL. This study provides experimental data showing that the torque to dissociation for all 100 tests was higher than the physiologically generated torque. Further studies will be needed to relate the GHJ forces and torque generated due to daily activities.

## Figures and Tables

**Table 1:** Mean force to dissociation for each implant.

Implant #	Implant	Mean Load (N)	95% CI for Mean Load (N)	Range (N)
1	Zimmer 1	2043 ± 235	1897–2188	1595–2349
2	Zimmer 2	1088 ± 166	985–1191	859–1367
3	Zimmer 3	1241 ± 148	1149–1333	1055–1486
4	Zimmer 4	1024 ± 248	871–1178	723–1464
5	Zimmer 5	2222 ± 454	1941–2503	1032–2222
6	Depuy	1285 ± 290	1105–1465	1025–2004
7	Exactech 1	1311 ± 230	1169–1453	1022–1677
8	Biomet 1	1371 ± 188	1255–1488	1157–1371
9	Biomet 2	2403 ± 236	2257–2549	1959–2730
10	Exactech 2	1142 ± 144	1053–1232	959–1441
Total Implant Average		1513 ± 508	1198–1828	1024–2403
ADL max = 578 N				

CI = confidence interval; ADL = activities of daily living.

**Table 2:** Implant group mean load to failure.

Manufacturer	*n*	Mean Load (N)	95% CI for Mean Load (N)	Range (N)
Zimmer	5	1524 ± 565	1028–2019	1024–2222
Depuy	1	1285	-	-
Exactech	2	1227 ± 119	1061–1392	1142–1311
Biomet	2	1887 ± 730	876–2898	1371–2403

CI = confidence interval.

**Table 3:** Mean rotation and torsional stiffness for each implant.

Implant #	Implant	Mean Rotation (°)	Mean Torsional Stiffness (N·m/°)
1	Zimmer 1	13.206 ± 3.278	4.483 ± 0.93
2	Zimmer 2	13.902 ± 4.035	4.684 ± 0.96
3	Zimmer 3	10.960 ± 2.051	5.854 ± 0.98
4	Zimmer 4	11.659 ± 2.360	5.821 ± 1.05
5	Zimmer 5	11.420	3.378
6	Depuy	12.022 ± 10.740	4.546 ± 2.26
7	Exactech 1	16.988 ± 18.125	3.691 ± 3.81
8	Biomet 1	7.300	8.441
9	Biomet 2	16.617 ± 16.128	5.631 ± 3.85
10	Exactech 2	7.65 ± 12.975	10.523 ± 6.81

**Table 4:** Mean torque to dissociation.

Implant #	Implant	Mean Torque (N·m)	95% CI for Mean Torque (N·m)	Range (N·m)
1	Zimmer 1	57.23 ± 8.55	49.73–64.73	46.84–69.35
2	Zimmer 2	62.07 ± 9.60	56.12–68.02	46.21–76.58
3	Zimmer 3	62.77 ± 6.38	58.82–66.72	52.49–75.39
4	Zimmer 4	65.98 ± 5.68	62.46–69.50	55.96–73.33
5	Zimmer 5	38.58	NA	NA
	Zimmer average	62.03 ± 8.63		
6	Depuy	32.96 ± 3.68	30.68–35.24	29.46–41.39
7	Exactech 1	11.58 ± 2.68	8.96–14.20	7.74–13.74
10	Exactech 2	21.97± 3.17	19.19–24.75	16.53–24.42
	Exactech average	17.25± 6.06		
8	Biomet 1	61.62	NA	NA
9	Biomet 2	52.33 ± 9.03	42.11–62.55	42.31–59.85
	Biomet average	54.65 ± 8.72		
	Total Implant Average	49.77 ± 19.07	29.85–63.57	7.74–76.58
	Physiologic estimate of torque	44.61 ± 14.12	40.00–49.22	22.37–75.79

CI = confidence interval.

**Table 5:** Mean displacement, torsional stiffness, and load for each implant.

Implant Number	Implant	Mean Displacement (mm)	Mean Tor Stiffness (N/mm)	Mean Load (N)
1	Zimmer 1	0.476 ± 0.039	4301.98 ± 494.54	2043 ± 234.86
2	Zimmer 2	0.897 ± 0.133	1218.88 ± 141.41	1088 ± 166.15
3	Zimmer 3	0.829 ± 0.161	1519.16 ± 160.65	1241 ± 148.29
4	Zimmer 4	0.828 ± 0.161	1246.33 ± 235.78	1024 ± 247.98
5	Zimmer 5	0.701 ± 0.152	3344.78 ± 937.01	2222 ± 453.63
6	Depuy	0.392 ± 0.134	3468.99 ± 812.43	1285 ± 290.30
7	Exactech 1	0.212 ± 0.078	6744.47 ± 2140.20	1311 ± 229.75
8	Biomet 1	0.343 ± 0.097	4107.84 ± 456.63	1371 ± 187.96
9	Biomet 2	0.794 ± 0.199	3181.07 ± 753.96	2403 ± 236.11
10	Exactech 2	0.562 ± 0.102	2072.87 ± 353.19	1142 ± 144.27

**Table 6:** The *p*-value correlations of three different taper conditions.

Implant #	Load Correlation			Displacement Correlation			Torsional Stiffness Correlation		
	Dry/ Bovine	Dry/ Water	Water/ Bovine	Dry/ Bovine	Dry/ Water	Water/ Bovine	Dry/ Bovine	Dry/ Water	Water/ Bovine
1	No	Yes	No	No	Yes	No	No	No	Yes
2	No	No	No	No	No	No	No	No	No
3	-	Yes	-	-	Yes	-	-	No	-
4	No	No	No	Yes	-	Yes	-	Yes	-
5	Yes	Yes	No	No	No	No	No	Yes	No
6	No	No	No	No	No	No	No	No	No
7	-	-	-	-	-	-	-	-	-
8	No	No	No	No	No	No	No	No	No
9	No	No	No	No	No	No	No	No	No
10	-	No	-	-	No	-	-	No	-

Yes = correlation exists; No = correlation does not exist.

## Data Availability

Not applicable.

## Appendix A

**Table A1:** Summary of test parameters obtained during experimental program.

		Pulling Strength (N)		Total Displacement (mm)		Axial Stiffness (N/mm)	
Implant Number	Number of Tests	Mean	Standard Deviation	Mean	Standard Deviation	Mean	Standard Deviation
1	10	2043	±234.86	0.476	±0.039	4301.98	±494.54
2	10	1088	±166.15	0.897	±0.133	1218.88	±141.41
3	10	1241	±148.29	0.829	±0.161	1519.16	±160.65
4	10	1024	±247.98	0.828	±0.161	1246.33	±235.78
5	10	2222	±453.63	0.701	±0.152	3344.78	±937.01
6	10	1285	±290.30	0.392	±0.134	3468.99	±812.43
7	10	1311	±229.75	0.212	±0.078	6744.47	±2140.20
8	10	1371	±187.96	0.343	±0.097	4107.84	±456.63
9	10	2403	±236.11	0.794	±0.199	3181.07	±753.96
10	10	1142	±0.102	0.562	±0.102	2072	±353.19.19
